# Distinct repeat motifs at the C-terminal region of CagA of *Helicobacter pylori* strains isolated from diseased patients and asymptomatic individuals in West Bengal, India

**DOI:** 10.1186/1757-4749-4-4

**Published:** 2012-05-25

**Authors:** Santanu Chattopadhyay, Rajashree Patra, Raghunath Chatterjee, Ronita De, Jawed Alam, T Ramamurthy, Abhijit Chowdhury, G Balakrish Nair, Douglas E Berg, Asish K Mukhopadhyay

**Affiliations:** 1 National Institute of Cholera and Enteric Diseases, Kolkata, 700010, India; 2 Centre for Liver Research School of Digestive and Liver Diseases, Institute of Post Graduate Medical Education & Research, Kolkata, India; 3 Washington University School of Medicine, Saint Louis, USA; 4 Division of Bacteriology, National Institute of Cholera and Enteric Diseases, P 33 CIT Road Scheme XM, Beliaghata, Kolkata, 700010, India

**Keywords:** Helicobacter pylori, CagA, Duodenal ulcer

## Abstract

****Background**:**

Infection with *Helicobacter pylori* strains that express CagA is associated with gastritis, peptic ulcer disease, and gastric adenocarcinoma. The biological function of CagA depends on tyrosine phosphorylation by a cellular kinase. The phosphate acceptor tyrosine moiety is present within the EPIYA motif at the C-terminal region of the protein. This region is highly polymorphic due to variations in the number of EPIYA motifs and the polymorphism found in spacer regions among EPIYA motifs. The aim of this study was to analyze the polymorphism at the C-terminal end of CagA and to evaluate its association with the clinical status of the host in West Bengal, India.

****Results**:**

Seventy-seven *H. pylori* strains isolated from patients with various clinical statuses were used to characterize the C-ternimal polymorphic region of CagA. Our analysis showed that there is no correlation between the previously described CagA types and various disease outcomes in Indian context. Further analyses of different CagA structures revealed that the repeat units in the spacer sequences within the EPIYA motifs are actually more discrete than the previously proposed models of CagA variants.

****Conclusion**:**

Our analyses suggest that EPIYA motifs as well as the spacer sequence units are present as distinct insertions and deletions, which possibly have arisen from extensive recombination events. Moreover, we have identified several new CagA types, which could not be typed by the existing systems and therefore, we have proposed a new typing system. We hypothesize that a *cagA* gene encoding higher number EPIYA motifs may perhaps have arisen from *cagA* genes that encode lesser EPIYA motifs by acquisition of DNA segments through recombination events.

## **Background**

The gastric pathogen *Helicobacter pylori* chronically infect more than half of human population. Although most infections are asymptomatic, 10-15 % of the *H. pylori* infected individuals develop chronic inflammation leading to atrophic gastritis, peptic ulcer as well as gastric adenocarcinoma [1,2].This pathogen was classified as a type I carcinogen by the World Health Organization in 1994. However, specific traits that enable a small proportion of this genetically diverse bacterium in the pathogenesis are poorly understood. Early studies showed that human convalescent sera collected from diseased patients significantly respond to a high molecular weight immunodominant bacterial protein known as CagA [3-5]. Subsequently, it was found that in western countries, *H. pylori* strains that express CagA and specific vacuolating cytotoxin (VacA) subtypes are significantly associated with the diseases [6-10]. Interestingly, the expression of these virulence markers does not seem to be similarly associated with the disease in Asian context [11]. Although a series of hypotheses were recently proposed the specific reason for the differences in disease outcome and the biological function of the CagA in gastric epithelium remained mostly unexplained.

The gene that encodes CagA is part of a ~40 kb horizontally acquired DNA segment in the *H. pylori* genome known as *cag* pathogenecity island (*cag*PAI) [8]. The *cag*PAI also contain genes encoding a type IV secretion system, to ensure efficient translocation of the CagA protein into the host epithelium [12-16]. After translocation, CagA multimerizes and undergoes tyrosine phosphorylation by Src family kinases to its tyrosine moiety, which resides in a repeat motif of five amino acids, glutamate-proline-isoleusine-tyrosine-alanine (EPIYA), located at the C-terminal region of the protein [17-20]. Phosphorylated CagA binds to the Src homology 2 (SH2) domain of the cellular phosphatase SHP-2, an important component of the cell cycle regulation pathway. Due to the intrinsic membrane tethering property of CagA the CagA-SHP-2 complex localizes to the plasma membrane of the host epithelium [21], leading to disregulation of the SHP-2. This event is necessary and sufficient to change the gastric epithelium to a transformed epithelium, which is characterized by altered cellular proliferation, migration and elongated cell morphology known as hummingbird phenotype [21,22]. The CagA also disrupts the tight junctions and causes loss of apical-basolateral polarity in epithelial cells [23,24].

The extent of biological activity of CagA is directly associated with the number of phosphorylation sites or the number of EPIYA motifs present at the C-terminal region of CagA [21,25,26]. Due to these repeats, molecular weight of the CagA protein varies from 128–148 kDa and the *cagA* gene shows extensive length polymorphism at the 3’ end. Several attempts were made to type the distinct CagA proteins and the *cagA* genes on the basis of its length polymorphism at the C-terminal and 3' ends, respectively [9,27-29]. The simplest method uses a PCR to amplify the 3’ end of the gene to distinguish the variations in the number and order of the EPIYA motif coding regions [30]. In this PCR, it was found that the “type A”, which gives ~642 bp amplicon and encodes three EPIYA repeats is the most prevalent CagA type. This type usually is not associated with the diseases. The “Type C”, which yields ~810 bp product, encodes four EPIYA repeats is associated with gastric adenocarcinoma in Japan. Although this nomenclature is useful in terms of assessing disease association of different CagA types, the validity of this typing system in terms of disease association and the prevalence of various CagA types have not been tested in different geographic regions [31,32]. Another typing method, which was used more often in recent studies addressing the biological function of CagA described the distribution of 5 EPIYA motifs as A-B-C-C-C in CagA of strain NCTC11637 [33]. Mutational analyses revealed that the first two EPIYA motifs (A and B positions) have little, if any, biological function while the other motifs (−C-C-C positions) are responsible for the CagA phosphorylation and CagA-SHP-2 complex formation, which leads to the hummingbird phenotype [33]. Moreover, the amino acid sequences, which are repeated among the third, fourth and fifth EPIYA motifs (−C-C-C) differ among strains isolated from West and East Asia. Accordingly, CagA was typed as Western-CagA-specific sequences (WSS) and East Asian-CagA-specific sequences (ESS) and this difference may account for differences in disease outcome between the two geographical regions.

The present study aimed to address a number of issues. First, we assessed the disease association with *cagA* 3' end variations in Indian *H. pylori* strains, where the incidence of gastric cancer is less but the incidence of duodenal ulcer is common. Second, the exact repeat units, which form the spacer regions among the EPIYA motifs, were determined to understand the precise mosaic structure of the CagA phosphorylation site repeat units. Finally, we have identified several new CagA types, which could not be typed with the existing nomenclature and this motivated us to propose a new typing system.

## **Results**

### **Prevalence of*cagA*types and clinical outcome**

**Figure 1:**
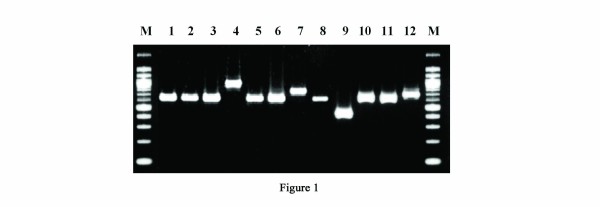
**Analysis of the 3' region of the*cagA*gene by PCR.** PCR products from a representative group of strains are shown. The sizes of the DNA fragments were confirmed after sequencing of the PCR products. Lane M, 100-bp molecular size markers.

**Figure 2:**
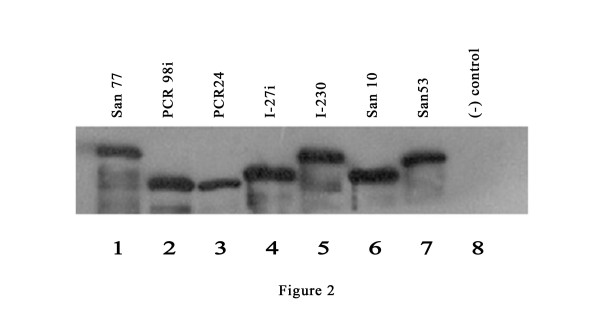
**Western blot analysis of*H. pylori*strains expressing representative CagA variants**. *H. pylori* strains were grown on brain heart infusion agar plates, harvested in PBS and subjected to SDS-PAGE followed by western blot analysis using anti-CagA antibody. Strain San 77, which carries CagA with five EPIYA motifs and gave ~810 bp amplicon in PCR using primer CAG1/CAG2 expressed CagA protein with highest molecular weight (lane 1). PCR98i and PCR24 which carry only two and single EPIYA motifs, gave ~550 bp and ~450 bp amplicon respectively in the same PCR expressed CagA proteins with much smaller size (lane 2 and 3). These two strains expressed CagA proteins, which are smaller than CagA expressed by strain San10 (lane 6) that carries 3 EPIYA motifs and gave ~642 bp amplicon, although I-27i (lane 4), which also carries two EPIYA motifsexpressed CagA protein with higher size than PCR98i (lane 2). Strain I-80, which carries no *cagA* gene, was used as negative control (lane 8).

Fifty-eight (31 of 40 duodenal ulcer patients (DU), 17 of 20 healthy volunteers (HV), 10 of 15 non-ulcer dyspeptics (NUD) and one from gastric adenocarcinoma patients (GC) of 77 *H. pylori* strains included in this study yielded a typical ~642 bp amplicon, designated as type A by Yamaoka et al. (1998) (Table 1). PCR using the same primer pair yielded ~756 bp amplicon corresponding to type B/D for 9 strains (4 from DU, 2 from HV and 3 from NUD) and ~810 bp amplicon corresponding to type C for 5 strains (3 from DU, 1 from HV and 1 from NUD) (Figure 1). Two strains from DU (I-27i and PCR 98i) and one strain from GC (PCR 93ii) gave unusual shorter amplicons (~550 bp). One strain isolated from NUD (PCR 24) yielded the shortest amplicon (~450 bp) encountered in this study (Table 1). None of these four short amplicons were described in the earlier typing system. No disease association was found with any of the CagA types. The expression of all CagA types was confirmed by Western blot using anti-CagA antidody (Figure 2). [The nucleotide sequences of the 3' end of *cagA* gene of 21 *H. pylori* strains were deposited in GenBank under the following accession numbers: EU089757 to EU089775, EU368669 and EU368670.]

**Table 1:** **Prevalence of*cagA*subtypes obtained in Kolkata and disease outcome**

***cagA*types**	**DU (N = 40)**	**Clinical status** **HV (N = 20)**	**NUD (N = 15)**
Type B/D	31 (77.5 %)	17 (85 %)	10(66.6 %)
Type B/D	4 (10 %)	2 (10 %)	3 (20 %)
Type C	3 (7.5 %)	1 (5 %)	1 (6.7 %)
New types	2 (5 %)	0 (0 %)	1 (6.7 %)

* Two strains isolated from gastric carcinoma patients were not shown in the table due to less sample size

### **Precise mosaic structure of the CagA**

Nine representative strains with *cagA* type A (5 from DU and 2 from HV and 1 each from CA and NUD), 6 strains with type B/D *cagA* (3 each from DU and HV), 2 strains with *cagA* type C (1 each from DU and HV) and 4 strains (I-27i, PCR24, PCR93ii and PCR98i) that yielded unusual amplicons were included in the sequence analysis. All the strains used in sequence analysis carried Western-CagA-specific sequences (WSS). These strains carried *cagA* genes capable of coding proteins containing 1–5 EPIYA motifs. Further analysis of the amino acid sequences predicted from nucleotide sequences of *cagA* 3' end revealed several distinct features present within EPIYA motifs.

**Figure 3:**
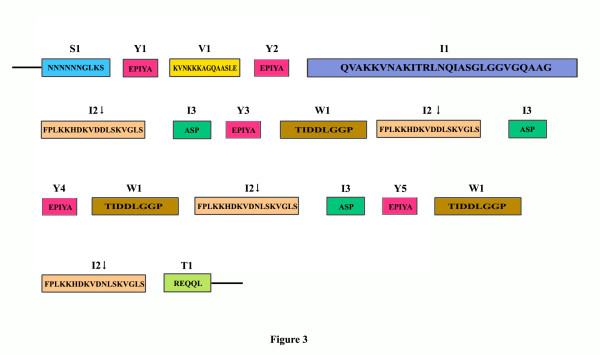
**Repeat motifs at the C-terminal region of CagA of strain San77.** Each motif was assigned with distinct colors. The first EPIYA motif is preceded by a stretch of 10 amino acids containing 6 successive asparagines (motif S1). First and second EPIYA motifs (Y1 and Y2) were connected by V1 region. The second EPIYA motif is followed by a motif called I1 and is never repeated while the sequence FPLKK---VGLS (motif I2) was repeated four times. In motif I2, a single amino acid replacement was noticed in third and fourth repeats as compared to first and second (shown with arrow). Third, fourth and fifth EPIYA motifs (Y3, Y4 and Y5) were always preceded and followed by I3 and W1 motifs. Remarkably, after fifth EPIYA motifs (Y5) and fourth I2 motifs there are no I3 motif present and are replaced by motif T1, which represents the ‘stop’ signal for this repeat region.

**Figure 4:**
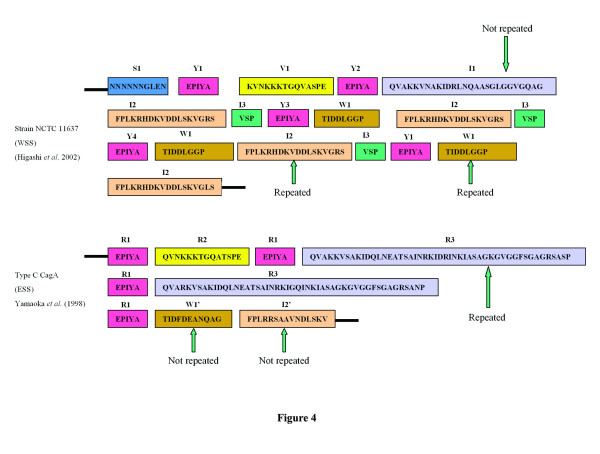
**Repeat motifs in Western (WSS) and Eastern (ESS) type CagA**. In WSS (NCTC 11637; reference 20**),** the I1 motif is not repeated, while the I2 and W1 motifs are repeated (shown with arrow). However, in ESS (see reference 42), the whole R3 region is repeated and the regions homologous to I2 and W1 are not repeated.

To illustrate the discrete repeat units, a CagA protein sequence (San 77) carrying the typical A-B-C-C-C type EPIYA motifs (and WSS), which produced ~810 bp amplicon with primers CAG1 and CAG2 was taken as model (Figure 3). These short repeat units were detected in CagA sequences of all the strains isolated from West Bengal and Western Countries (those carrying WSS) (Figure 4). The first EPIYA motif (termed as Y1 in this study to denote tyrosine phosphorylation site) in this CagA primary structure was preceded by a 10 amino acids motif (S1) containing a stretch of 6 asparagines (Figure 3). Irrespective of CagA types, this motif was detected in all CagA proteins having WSS (Figure 4).

**Figure 5:**
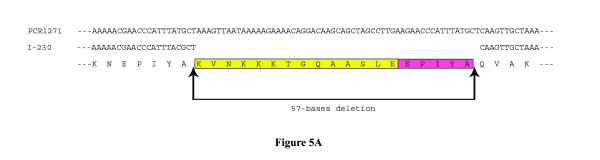
**(a) Alignment of nucleotide sequence of*cagA*3' end repeat region of strain PCR 127i and I-230.** Predicted amino acid sequence was given at the bottom of the figure and motifs are represented as different colors. A 57 base deletion was observed in strain I-230, which results in loss of 19 amino acids. These 19 amino acids include V1 and an EPIYA motif (Y2). (**b**) Identification of deletions observed in the repeat region at the C-terminal of CagA of *H. pylori* strains isolated from India. Each motif was assigned a color and the regions deleted were shown in dashed line.

The first and the second EPIYA (termed as Y2) motifs are separated by a motif named V1 that contains 13 amino acids. The V1 motif was found in all CagA sequences included in this study that carry A-B-C or A-B-C-C or A-B-C-C-C type EPIYA patterns. In agreement to the previous report, no type D CagA carried similar motif. Our analysis indicated that the absence of V1 and Y2 in type D CagA is due to a specific deletion of this locus (Figure 5b). Detection of several other deletions in several other CagA types further supported this connotation (Figure 5b).

Our analysis also indicates that the spacer regions of third, fourth and fifth EPIYA (Y3, Y4 and Y5) motifs contain several short discrete repeat motifs. The I1 motif was present immediately after the Y2 and was never reappeared (Figure 4). This stretch was followed by I2 and I3, which was present just before the Y3. As shown in Figures 3 and 4, I2 and I3 were repeated independent of each other but always maintained their respective positions and I2 was repeated one more time than I3.

The Y3 is followed by another distinct motif, W1, which is followed by I2 and I3 in A-B-C-C and A-B-C-C-C type CagA. However, in A-B-C type CagA (Figure 5B, strain PCR29i), though W1 and I2 were always present, I3 was replaced by another motif, T1. In A-B-C-C-C type CagA, I3 is repeated 3 times along with EPIYA motifs (at C positions) and after the last EPIYA at C position (Y5), I3 was replaced by T1 (Figure 5b). The comparative analysis of all the CagA primary structures revealed that T1, which must be present after I2 (and in the place of I3) indicates the termination of this repeat sequences and after T1 no EPIYA motif was detected in any CagA primary structure.

The differences in PCR product sizes observed between type C strains isolated from Japan and West Bengal were because the intact R3 region is repeated twice in ESS carrying CagA reported for Japanese *H. pylori* strains, while CagA found in West Bengal (and also for all WSS carrying CagA), only I2 and I3 is repeated but not I1. Another important feature of WSS carrying CagA is W1 (TIDDLGGP) is repeated after all EPIYA motifs at C positions (Y3, Y4, Y5), while the sequence, which replaces this motif in ESS CagA (TIDFGEANQAG) is present only once, after the last EPIYA motif (Y3 for A-B-C type, Y4 for A-B-C-C and Y5 for A-B-C-C-C type). The motif in ESS CagA (FPLRRSAAVRDLSKVGLS), which is homologous to I2, is also present only once, immediately before T1.

### **Description of new CagA types and development of new typing system**

Our sequence analyses identified several new CagA primary structures, which could not be typed by previously suggested typing system. The strains that gave unusual ~550 bp amplicons (PCR93ii and PCR98i) were found to carry unique CagA primary structures. Interestingly, in these two CagA primary structures, I3 and W1 regions were missing. The entire sequence between the Y2 and the T1 was also absent, possibly due to a deletion event (Figure 5B). In other words, for these two CagA types, EPIYA motifs were present at A and B positions only, while there was no EPIYA motif at C position [33]. However, the strain I-27i, which yielded similar ~550 bp amplicon contains entirely different CagA primary structure. In this CagA, V1 and the adjacent EPIYA (Y2) at B position were absent (Figure 5b). The shortest amplicon (~450 bp) was obtained in this study with strain PCR24. In this CagA, the S1 was followed by only one EPIYA motif and then a big deletion was detected as several repeat units such as V1, Y2, I1, I2, I3 and Y3 were entirely missing. Since previous typing systems failed to type these CagA primary structures we suggest a new typing system (Table 2).

**Table 2:** CagA primary structures commonly observed in India and in Western countries and the new typing system.

**CagA primary structures**	**Types described by Yamaoka*et al.*(1998)**	**Types described by Higashi*et al.*(2002)**	**New typing system (this study)**	**Examples in this study**
S1Y1V1Y2I1I2I3Y3W1I2T1	Type A	A-B-C	Type 1	PCR29i
S1Y1V1Y2I1I2I3Y3W1I2I3Y4W1I2T1	Type C	A-B-C-C	Type 2	San53
S1Y1V1Y2I1I2I3Y3W1I2I3Y4W1I2I3Y5W1I2T1	Type ?	A-B-C-C-C	Type 3	San77
S1Y1I1I2I3Y3W1I2I3Y4W1I2T1	Type D	A-C-C	Type 4	I-230
S1Y1I1I2I3Y3W1I2T1	Type ?	A-C (?)	Type 5	I-27i
S1Y1W1I2T1	Type ?	A (?)	Type 6	PCR24
S1Y1V1Y2I1I2T1	Type ?	A (?)	Type 7	PCR98

Some of these types could not be addressed with previous systems and termed as “type ?”

### **Natural insertion and deletions**

Our analyses suggest that the distinct repeat motifs could be present in the form of either deletion or insertion. To further confirm this hypothesis, CagA from 2 strains (San53 and PCR29i) could be taken as examples. As shown in Figure 5, these two primary structures revealed that only a portion of CagA sequence (W1I2I3Y4, shown in underline and italics) in strain San53 was absent in CagA primary structure of strain PCR 29i. In other words, if the region W1I2I3Y4 could be inserted to CagA primary structure of strain PCR 29i, between Y3 and W1 (shown in bold), both these strains would have similar CagA.

Analysis also revealed that strains (eg. I-230) carrying previously described type D CagA actually has a deletion of V1Y2 regions and if this region were inserted between Y1 and I1 (shown in bold) they would have a CagA similar to that of type C (Figure 5a). Even more interesting observations were obtained while analyzing CagA primary structure of strain I-27i where region W1I2I3Y4 was deleted. Although this strain gave an amplicon closer to type A by PCR using primers CAG1 and CAG2, primary structure analysis revealed that this CagA was closely related to type D and insertion of W1I2I3Y4 between Y3 and W1 (shown in bold) could make it exactly similar to type D CagA (Figure 5b).

**Figure 6:**
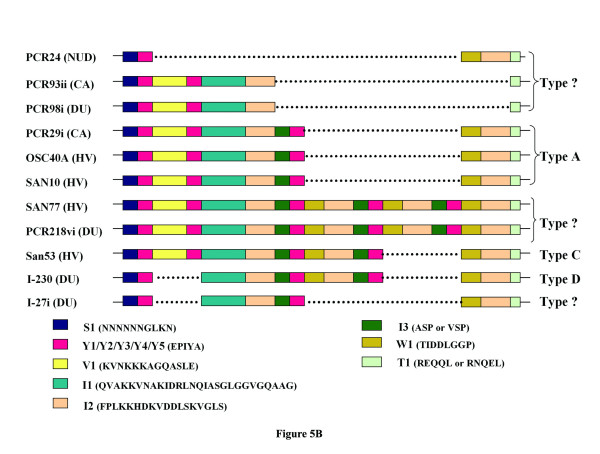
**Schematic representation of various types of deletions or insertions among the CagA isolated from WB, India.** The segments deleted or inserted are mentioned along with the arrows. The regions where the insertions have occurred are shown in bold and larger front. For type 2 and type 4 CagA, the V1Y2 motifs were inserted within Y1 and I1 and shown in bold, larger front size and also with underscore. As shown in the diagram, almost all the types could be converted to the other types with the occurrence of insertions or deletions.

Further larger deletions in CagA were noticed in strains PCR93ii and PCR98i. Both these strains had a deletion of I3Y3W1I2 between I2 and T1 (shown in bold). Strain PCR24, which carry only one EPIYA motif, had the largest deletion. The entire region between Y1 and W1 (shown in bold) was deleted in this CagA and it was not possible to predict from which type of CagA it has evolved through deletion. On the other hand, it is possible that this CagA bears the core unit and the other complex CagA structures have evolved from it (Figure 6).

### **Putative recombination breakpoint analysis**

**Figure 7:**
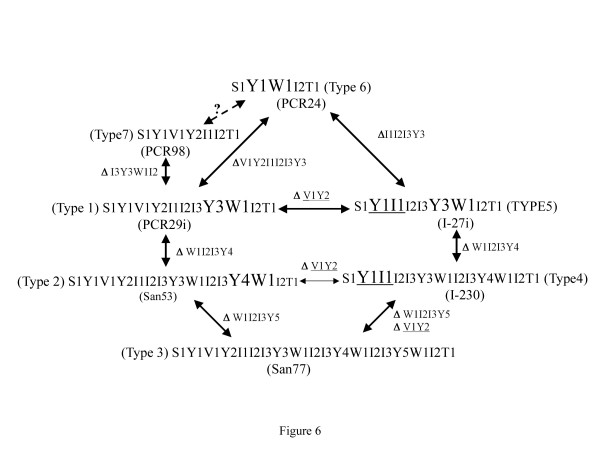
**Recombination breakpoint analysis of aligned sequences of 3' end of*cagA*of 11** ***H****. pylori* strains along with the annotated *H. pylori* P12 strain using TOPALi. Numbers in bold above and below the peaks indicate the putative recombination breakpoint positions. The dotted horizontal line is the 95 % significance point of *Dss*. Start of the alignment of 3’ end sequences of *cagA* corresponds to the 2638 bps from the start of the *cagA* gene (CP001217) of *H. pylori* p12 strain.

TOPALi is graphical analysis software that uses statistical analysis to detect the presence of possible recombination sequences and gives an approximate location of the breakpoints within DNA multiple alignments [34]. As described in the methods, 3' end sequences of *cagA* gene of 19 *H. pylori* sequences were aligned, and the putative recombination breakpoints were analyzed by TOPALi (Figure 7). Using TOPALi the recombination breakpoints are predicted at 290, 331, 381 and 447 bp. The sequences between the putative breakpoints 291 and 331 are between Y3 and Y4 motif, and that between 381 and 447 are between Y4 and Y5 motif. The first hypervariable region, predicted using TOPALi, includes Y3, W1 and parts of second I2 motif. The second hypevariable region includes I3, Y4, W1, I2, I3, Y5 and parts of corresponding W1 motif. The predicted recombined regions in the 3' end sequences of *cagA* gene may account for the sequence variation of *cagA* gene. This analysis strengthens the hypothesis that different complex types of *cagA* genes are evolved from the CagA that bears the core unit due to the gene conversion at the border of the hypervariable regions.

**Figure 8:**
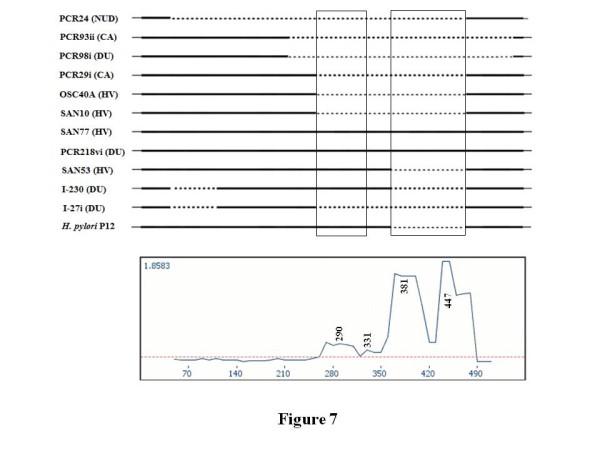
**Secondary structure cartoon of San77 predicted using Discrimination of protein Secondary structure Class (DSC) in Discovery studio (Accelrys, USA).** Helices are colored in red, strands are in blue, and coils are in beige. Tyrosine residues in first and second EPIYA motifs are within the alpha-helical region. Tyrosine residues in third, fourth and fifth ‘EPIYA’ motifs are within the beta-strands.

Disease associations of the *H. pylori* strains are reported to be strongly correlated with the presence of this complex domain architecture with increasing number of repeat sequences containing EPIYA motifs of CagA gene [25,26,30,33]. To assess the possible role of the presence of this motif, we predicted secondary structure of San77, which comprises of the entire five EPIYA motifs (i.e., Y1Y2Y3Y4Y5), using Discrimination of protein Secondary structure Class (DSC) module of Discovery studio (Figure 8). The tyrosine residue in the first and second EPIYA motifs are being contained in the α-helical conformation, whereas that of the Y3, Y4 and Y5 are contained in the beta strand conformation preceding a coil spanning from end of I2 to I3 motif. One may hypothesize that the presence of this tyrosine residue in the α-helical conformation may inhibit the phosphorylation of the tyrosine residue by making it less accessible. In contrast, the tyrosine residues in the Y3, Y4 and Y5 motifs might be more exposed and accessible to be phosphorylated due to their presence in β-strand conformation and the presence of preceding loop conformation. A similar observation was also reported in an earlier study [35]. It could readily explain the reason why CagA proteins having more 34-amino acid repeat sequences undergo greater tyrosine phosphorylation and hence exhibit increased SHP-2 binding and induce greater morphological changes.

## **Discussion**

The length polymorphism observed at the 3' end of *cagA* gene of *H. pylori*, which results in variation in the number of phosphorylation sites of the encoded protein, is of great interest in recent times since higher number of the phosphorylation sites in CagA was described to be associated with stronger biological function and disease manifestation [25,26,30,33,36]. Several attempts have been made to type this virulence marker considering the variability of the phosphorylation sites (EPIYA) and certain types seem to be associated with the clinical outcome [9,27-30,33,37,38]. However, in India, where ~85-90 % of *H. pylori* strains carry the *cagA* gene [32], data that relate the polymorphism at C-terminal end of the CagA and the clinical status of the host are scanty [39].

We have found that previously described type A (type 1, in our proposed nomenclature), which is the most prevalent CagA type in other parts of the world, was also detected in West Bengal. However, there was no disease association with any of the previously described CagA types. Infection with *H. pylori* strain carrying CagA with higher number of EPIYA motifs (A-B-C-C-C), which was described to be associated with gastric cancer, was also isolated from healthy individuals. The expression of A-B-C-C-C type CagA in *H. pylori* strain isolated from HV was also confirmed by western blot analysis. In addition, strains isolated from healthy individuals and those from patients with overt diseases like peptic ulcer or gastric adenocarcinoma showed identical amino acid repeat motifs in the CagA.

Previous reports indicate that the amino acid sequences among the EPIYA motifs vary between Western CagA and East-Asian CagA [25,26,33]. The sequence FPLKRHDKVDDLSKV found in western type CagA (WSS) is replaced by sequence KIASAGKGVGGFSGA in East-Asian type CagA (ESS) and this variation in amino acid sequences might be the reason behind the high prevalence of gastric cancer in Japan as compared to the West [25,30,33]. Interestingly, in this study we have found that in Indian context, where the prevalence of duodenal ulcer is more common than gastric cancer, all the strains used for sequence analysis carried western type CagA. This finding is in agreement with previous report which describes that the conserved sequences of *cagA* 5’ end region in *H. pylori* strains isolated from Calcutta form cluster with *cagA* sequences of *H. pylori* strains isolated from western countries and differ significantly from *cagA* sequences found in East-Asia.

Further analysis of different CagA primary structures revealed that the C-terminal repeat region is actually more discrete from the previously proposed models of CagA variants. For example, from this study it is apparent that the R3 region (originally described by Yamaoka et al for ESS), for WSS CagA, contains 3 distinct repeat regions viz. I1, I2 and I3. The CagA primary structure model proposed by Yamaoka et al. (1998) describes that the R3 region is present between second and third EPIYA motifs and also between third and fourth EPIYA motifs. However, our analysis with strains isolated from West Bengal and Western countries showed that although intact R3 region (combination of I1-I2-I3) is present between second and third EPIYA motifs (Y2 and Y3 respectively), in type C (type 2, in the proposed nomenclature) only I2 and I3 is present between third and fourth EPIYA motifs (Y3 and Y4 respectively) and I1 is not repeated. The 34 amino acids repeat region described by Azuma et al. (2004), which is present within third to fifth EPIYA motifs (Y3 to Y5) can be divided as smaller units (Figure 4). The amino acid sequence analysis of CagA in the current study showed that this region includes four distinct, short repeat motifs containing I2, I3, W1 (TIDDLGGP) and Y (EPIYA). For example, in A-B-C-C-C type CagA, I2 is repeated four times, while I3 and W1 repeated three times and after the last EPIYA motif present in position C (Y5), I3 is replaced by T1 motif. We have noticed that T1 motif represents the termination of this repeat region for all CagA types and after this T1 motif, no EPIYA motif was detected.

Our analysis revealed that the EPIYA motifs as well as the spacer sequence units among the EPIYA motifs are present as distinct insertions and deletions. For example, between the first two EPIYA repeats, the identified region named as R2 (V1, in the proposed nomenclature) by Yamaoka et al. (1998) for type C (type 2, in the proposed nomenclature) CagA is absent in type D (type 4, in the proposed nomenclature) CagA, although the remaining motifs are identical. This suggests that the absence of R2 region in type 4 strains is possibly resulted from a deletion. Many other deletions or insertions, which we have shown in our present report, might have occurred due to extensive genetic rearrangements or recombination events during the evolution. In *Neisseria gonorrhoeae* for example, the pilin genes show extensive variation at the 3' end while the 5' end remain relatively conserved and undergo recombination in order to generate antigenic variation of pilin expression [24]. This reassortment of pilin genes in this organism occurs possibly by two distinct mechanisms [40]. Both piliated and revertible P- cells of *N. gonorrhoeae* are competent for in vitro DNA uptake during autolysis. This mechanism suggests that pilin antigenic variation occurs through transformation by exogenous DNA liberated from lysed cells within a population [41]. In addition to this, transformation-mediated recombination and intragenomic reciprocal recombination also contribute to the pilin antigenic variation [40]. Likewise, *H. pylori* is also naturally competent [42], undergoes autolysis [43] and the frequency of recombination in this species is very high [44]. This indicates that transformation-mediated recombination might be responsible for the extensive variations observed at the 3' end of *cagA* gene. Moreover, the *cag*PAI contains genes that code for a putative type IV pilus, which is involved in CagA translocation to the host cell could also be involved in the DNA uptake [12,14-16]. Intragenomic reciprocal recombination between two *cagA* loci of the same chromosome is also possible as multiple *H. pylori* strains were isolated from single gastric niche, which had identical DNA fingerprint with polymorphism only at the 3' end repeat region of *cagA* (unpublished data). It is important to mention here that the CagA, apart from SHP2, interacts with several other proteins, which are important for cell cycle regulation and EPIYA motifs, in phosphorylated as well as unphosphorylated form, are possibly the active sites of CagA [23,45,46]. Therefore, recombination in these sites is possibly important for *H. pylori* to manifest different levels of biological activity in order to survive in human gastric mucosa- an environment, which changes often with food habits and other lifestyle of the host. Variation in these repeats should also result in variation in three-dimensional structure of CagA protein. These structural changes, apart from the variability in biological function in terms of interfering with several signaling pathways, might result in minute changes in antigenicity, which is possibly necessary for the pathogen to escape host immune surveillance, considering the CagA is a highly immunodominant protein. Therefore, *H. pylori* strains, under continuous selection pressure, may recombine with high efficiency during prolonged colonization in gastric mucosa resulting emergence of different CagA types.

## **Conclusions**

In Summary, we have identified seven CagA types and several of them are being reported for the first time including a CagA type, which carry a single EPIYA motif. These new CagA structures could not be typed by the existing systems and therefore, we have proposed a new typing system. Although this typing system is developed by analyzing CagA primary structures of *H. pylori* strains isolated in West Bengal, India, we have noticed that it is suitable to type all CagA primary structures having WSS detected in other parts of the world. Previously, it was shown by Higashi et al (2002) that tyrosine phosphorylation at C position is necessary and sufficient for CagA-SHP2 complex formation and the tyrosine residues at A and B positions have little functional significance. We have identified two strains, which carry CagA with EPIYA motifs present at A and B positions (Y1 and Y2 respectively) only but lack the same motif at C position (Y3) as this region is found to be absent due to natural deletion. Interestingly, one of these two strains was isolated from a patient with gastric adenocarcinoma. Moreover, we have found that the *H. pylori* strain (San 77) isolated from healthy individual can express A-B-C-C-C type CagA. Thus, our data strongly indicate that carrying a particular type of CagA is not the only determinant for the disease outcome especially in the developing countries like India, where multiple infections with different CagA primary structures are possible (unpublished data). We believe that CagA status of the infected *H. pylori* strains as well as other factors must contribute to cause disease and host genetic traits are the most important among them.

## **Materials and methods**

### **Patient samples and*H. pylori*strains**

A total of 77 *H. pylori* strains, 40 from DU, 20 from HV, 15 from NUD and 2 from GC were isolated as described earlier [11,32]. In brief, these individuals underwent a non-sedated upper gastrointestinal endoscopy (GIF XQ 30, Olympus Optical Company, Japan) under topical lignocaine anesthesia at the Hospital of the Institute of Post Graduate Medical Education and Research in Kolkata, India. Written informed consent was obtained from each of these individuals as per the recommendations of the Ethical Committee of the Institute of Post Graduate Medical Education and Research and the study was ethically approved by the committee. Gastric biopsy specimens were transported in 1 ml of Brucella broth (Becton Dickinson, Sparks, MD, USA) containing 15 % of glycerol in cold condition for culture at the Helicobacter unit of the National Institute of Cholera and Enteric Diseases. *H. pylori* was isolated from biopsy specimens, sub-cultured and preserved at −70°C in brain heart infusion (BHI) (BD) containing 15 % glycerol as described earlier [11,32].

### **Characterization of*H. pylori*strains by PCR**

*H. pylori* genomic DNA was extracted by CTAB (hexadecyltrimethyl ammonium bromide) method [47] from confluent lawn of bacterial culture on BHI agar (BD) plate. All the strains included in this study carried *cagA* gene as determined previously by PCR using primers designed from 5' end conserved regions [32,48]. Genomic DNA extracted from these strains was used to amplify 3' end repeat region of *cagA* with primers CAG1 (5'–ACCCTAGTCGGTAATGGGTTA-3') and CAG2 (5'–GTAATTGTCTAGTTTCGC-3') [30].

### **Nucleotide sequencing of PCR products and sequence analysis**

PCR products were purified by QIAquick PCR purification kit (Qiagen GmbH, Hilden, Germany) and used directly as a template for nucleotide sequencing. Both strands of DNA were sequenced with the Big-dye terminator cycle sequencing kit (Applied Biosystems, Foster city, CA) according to the manufacturer’s instructions, using an ABI PRISM 310 sequencer (Applied Biosystems). The nucleotide and deduced amino acid sequences were edited and compared after aligning using SeqMan and EditSeq program of DNASTAR (DNASTAR, Inc). Multiple alignments were done by using ClustalW in MegAlign software (DNASTAR, Inc.). Finally, the discrete motifs present at the C-terminal end of the CagA were identified by manual analysis.

### **Western blotting**

*H. pylori* strains cultured on BHI agar were harvested in phosphate-buffered saline (PBS), and subjected to SDS-PAGE followed by transfer to nitrocellulose membrane. Proteins immobilized on membrane were incubated with anti-CagA antibody (Austral biologicals, CA) diluted 1:4000 in Tris buffer saline containing 3 % bovine serum albumin and 1 % tween-20 for 1 hr, washed and incubated with anti-rabbit antibody (Santa Cruz Biotech., CA) for 1 hr. After washing immunoreactive components were visualized by ECL detection system (Amersham).

### **Nucleotide sequence accession numbers**

The nucleotide sequences of the 3' end of *cagA* gene of 21 *H. pylori* strains were deposited in GenBank under the following accession numbers: EU089757 to EU089775, EU368669 and EU368670.

### **Sequence and putative recombination breakpoint analysis**

To predict the *cagA* recombination sites, the 3' end sequences of *cagA* gene of 21 *H. pylori* strains were aligned in ClustalW [49] and some manual corrections were made to achieve accurate alignment. The aligned sequences were analyzed by the TOPALi software. The detailed description of the algorithm of TOPALi software was available elsewhere [34,50,51]. Briefly, the pairwise distance matrix of the aligned sequences was calculated using the Jukes-Cantor nucleotide substitution model. A window *i* of user-defined length was moved forward and backward along the sequence alignment at a certain stepwise intervals. For each window *i*, the difference of sums of squares of forward (*Dss*^*i*^_*F*_) and backward (*Dss*^*i*^_*B*_) windows were calculated. The corresponding *Dss* value was obtained using from the maximum of *Dss*^*i*^_*F*_ and *Dss*^*i*^_*B*_. *Dss* values were plotted for each window. A set of adjacent large *Dss* values forming a significant peak suggested a putative recombination breakpoint.

## **Competing interests**

The authors declare that they have no competing interests.

## **Authors’ contributions**

SC and AKM conceived of the study, GBN, TR and DEB participated in the design of this study; AC, SC and RP coordinated collection of specimens, maintenance of clinical data and management of patients; SC, RP, RC, RD and JA carried out bacterial isolation and bacterial sequencing and sequence analysis using different soft wares. All authors read and approved the final manuscript.
